# Neutrophil gelatinase–associated lipocalin as an immunomodulator in endocrine hypertension

**DOI:** 10.3389/fendo.2022.1006790

**Published:** 2022-10-25

**Authors:** Patricio Araos, Cristián A. Amador

**Affiliations:** ^1^ Laboratorio de Fisiopatología Renal, Instituto de Ciencias Biomédicas, Universidad Autónoma de Chile, Santiago, Chile; ^2^ Facultad de Medicina y Ciencia, Universidad San Sebastián, Santiago, Chile

**Keywords:** NGAL, endocrine hypertension, aldosterone, immune system, mineralocorticoid receptors

## Abstract

In recent studies, primary aldosteronism (PA) has been reported as the most common etiology for secondary hypertension of endocrine origin, accounting for approximately 10% of cases. In PA, excess aldosterone production can lead to deleterious effects at the cardiovascular (CV) and renal levels by activating mineralocorticoid receptors, which involves an increase in pro-inflammatory and pro-fibrotic mediators. Among these mediators, neutrophil gelatinase–associated lipocalin (NGAL), a secretion glycoprotein belonging to the lipocalin superfamily, has been closely linked to CV and renal damage in several pathological conditions. Because NGAL can be detected in biofluids such as plasma and urine, it has been proposed as a damage biomarker for target tissues and has also been studied for its role in hypertension and associated with PA. NGAL is produced by many different cell types, can be carried on extracellular vesicles, and is modulated by microRNAs, which would support its use as a biomarker for endocrine hypertension due to PA. Over the last decade, studies have shown that NGAL is necessary for the development of aldosterone-induced hypertension and that is associated with end-organ damage. In addition, it has been proposed that some mechanisms are dependent on the activation of immune cells, such as dendritic cells and macrophages, where the release of specific cytokines (i.e., interleukin [IL]-23) or chemokines (i.e., CCL-5) induced by aldosterone would depend on NGAL. Subsequently, this activates the T helper (Th) lymphocytes, such as Th_17_ and Th_2_, resulting in CV and renal fibrosis due to the high aldosterone levels. Although the immune system has been closely associated with essential hypertension, its participation in endocrine hypertension has not been fully elucidated. This review discusses the link between NGAL and endocrine hypertension, particularly in the context of PA, and their possible regulators and mechanisms, with a focus on its role as an immunomodulator.

## Introduction

Arterial hypertension (AH) affects 31.1% of the global population (1). Its etiology is multifactorial, and many cases are classified as essential AH because their precise origin is unknown. Approximately 10%–15% of hypertensive patients have secondary AH, with renal arterial disease and endocrine AH as the most common etiologies. Primary aldosteronism (PA) is the most common etiology for endocrine AH (2).

Neutrophil gelatinase–associated lipocalin (NGAL), also known as lipocalin-2 (Lcn2) or 24p3, is a 25-kDa secretion protein belonging to the lipocalin superfamily, which can bind siderophores for iron import to different cell types (3). Although its expression may vary in some physiological conditions (4, 5), a significant increase in NGAL levels has been closely related to several renal and cardiovascular (CV) disorders, involving the activation of the mineralocorticoid receptors (MR). Thus, NGAL has emerged as a possible biomarker for acute and chronic sub-inflammatory conditions, including PA.

Here, we present several conditions that can modify NGAL abundance depending on aldosterone (Aldo) levels. Our discussion will mainly focus on the mechanisms where NGAL may function as a modulator of the immune response driving tissue fibrosis, in hopes of providing new insight into its role as a biomarker for PA.

## Endocrine arterial hypertension and primary aldosteronism

PA is clinically defined as inappropriate synthesis and secretion of Aldo, independent of the renin–angiotensin system, that cannot be suppressed by sodium loading (6). The prevalence of PA varies depending on the diagnostic criteria, but approximately 10% of patients with AH present PA (7), and its prevalence may be increased in patients with resistant AH (8). Patients with PA have a higher risk of CV and renal complications, as well as a higher risk mortality as compared to those with essential AH (9–11). Excess Aldo production favors MR activation in different cell types, which can increase the risk of morbidity and mortality (9, 12) even in normotensive patients (13). Thus, increased MR activity due to PA is seen in AH and in target organ damage.

Current guidelines recommend using the Aldo to renin ratio for PA analysis (14). However, sensitive technology (e.g., liquid chromatography–mass spectrometry) is required for quantification because of the low plasma concentrations of these components. This relationship may also be modified by different factors (15). Therefore, early identification of PA, with emphasis on Aldo target tissues and cells, both represents a challenge related to its diagnosis (16) and is also relevant for the stratification of risk related to the pro-inflammatory and pro-fibrotic that can be caused by Aldo–MR interaction in different cell types (17).

NGAL is a sensitive target gene to the complex Aldo–MR, which has allowed to consider it as a biomarker for CV and renal disease associated with PA (18, 19). Moreover, recent findings suggest that extracellular vesicles (EVs) isolated from liquid biopsies may carry NGAL, representing a new diagnostic tool for PA (20).

## NGAL as a biomarker of renal damage and arterial hypertension

Kjeldsen et al. initially described NGAL in human neutrophils (21, 22), where it is expressed in forms of mRNA and protein change based on the granulopoiesis stage (23). However, NGAL can originate from many sites: the hematopoietic organs (24); adipocytes; hepatocytes (25); neoplastic cells; lungs (26); mammary glands and uterus (4); neurons; and endothelial, cardiac, and smooth muscle cells (27).

Recently, we characterized the relative abundance of NGAL in different mouse immune cells. We observed that its expression in CD11c^high^ antigen-presenting cells (APCs) is higher than that in B and T lymphocytes (28). This observation is relevant considering that these immune cells can potentially promote AH (18) and end-organ damage associated with AH (29).

In the kidney, NGAL expression has been described in specific nephron regions augmented in different experimental and clinical settings of renal dysfunction, suggesting that NGAL is a specific biomarker for kidney injury (30–33). In particular, NGAL increases considerably in response to ischemic or obstructive renal disease in mice (31, 34, 35), suggesting a direct correlation between inflammatory processes and oxidative stress in the development of kidney disease. Furthermore, Viau et al. showed the prevention of tubular dilation and glomerular and tubular fibrosis in an experimental model of chronic kidney disease (CKD) in NGAL knock-out (KO) mice (36), indicating its critical role.

In terms of NGAL’s potential as a biomarker, studies have shown that high urinary NGAL urinary levels of NGAL (uNGAL) levels are directly associated with tubulointerstitial fibrosis and tubular atrophy in patients and experimental animals. In contrast, it is inversely correlated with the glomerular filtration rate in kidney disease (36, 37). Over the last few years, studies have proposed that early identification of uNGAL or serum NGAL (sNGAL) would be helpful not only in the early diagnosis of kidney disease (37–39) but also in tracking the progression of acute kidney injury (31) and its transition to CKD in real time (40). Thus, using NGAL as a biomarker in the early stages of renal damage could be crucial for the prognosis of patients progressing to CKD (41).

Because CKD and AH are closely linked, several studies published during the last decade have considered using sNGAL and/or uNGAL as biomarkers to determine the CV and renal risk in patients with AH. For instance, uNGAL levels (normalized to urine creatinine) were found to correlate with systolic blood pressure (SBP) and diastolic blood pressure (DBP) in a study of 100 healthy volunteers from Hong Kong (42). Similarly, Wu et al. demonstrated that sNGAL was significantly associated with SBP and DBP in a cohort of 707 patients, comprising those with CV disease and those who were CV disease–free (43). Malyszko et al. showed that uNGAL and sNGAL levels in hypertensive patients were higher than in normotensive and healthy volunteers (44). Because the NGAL levels in serum and urine may be modified by a patient's renal function, the increase in sNGAL and uNGAL levels may also be affected, considering the differences in renal function among the study participants. Finally, Lindberg et al. revealed that plasma NGAL levels were positively correlated with SBP and neutrophil count and inversely correlated with renal function (45). All of these studies defined AH as SBP > 140 mmHg without specifying its etiology. The origin of the increase in uNGAL and sNGAL levels observed in endocrine AH therefore remains to be clarified. However, the origin of these increased levels in the context of PA is well understood, and the mechanisms underlying this phenomenon will be discussed in the following sections.

Recently, the use of NGAL carried on EVs as a biomarker in hypertensive patients has also been considered. Several studies in renal transplantation have demonstrated that exosome cargo obtained from urine involves NGAL in the forms of mRNA (46) and protein (47). Other studies in central and peripheral inflammatory conditions have shown NGAL expression in EVs isolated from biofluids such as plasma (48) and saliva (49). In the case of AH, Barros et al. showed that alpha-1-acid-glycoprotein (AGP1, also known as orosomucoid-1) is upregulated in urinary EVs from hypertensive patients compared with those of normotensive patients (50). AGP1 is an inflammation-sensitive plasma protein that is increased in patients with higher CV risk (51) and is related to lipocalins, including NGAL. However, NGAL upregulation in the EVs of hypertensive patients remains to be explored.

## NGAL and endocrine arterial hypertension

To date, the hormonal regulation of NGAL expression associated with AH has been poorly investigated. In this context, excess glucocorticoid production due to an endogenous or exogenous origin induces AH (52) by activating the glucocorticoid receptors (GR) in peripheral tissues (53). In particular, the GR in vascular smooth muscle cells has been considered crucial for increasing blood pressure during glucocorticoid excess (54). This evidence is relevant, considering that the GR at the CV level modulate NGAL abundance during dexamethasone treatment (55). In addition, an experimental study in rats showed that 2-week oral administration of corticosterone increased NGAL protein expression in the glomeruli and renal tubule by 75% and 30%, respectively (56).

In terms of other “non-classic” tissues (or cells) in AH, Vizzardelli et al. reported that glucocorticoids have a synergic effect on lipopolysaccharides involved in NGAL production in dendritic cells (DCs) (57), which are the principal APCs involved in AH. Because high glucocorticoid levels can also activate the MR, it is worth mentioning that some of the effects described could occur through this receptor (58). This hypothesis is supported by a recent study that reported that hypertensive patients diagnosed with non-classic apparent mineralocorticoid excess plus PA presented higher NGAL plasma levels than those with PA only (59). The regulation of NGAL by the glucocorticoid–MR complex in other immune cell types in endocrine AH remains to be explored further.

NGAL modulation due to MR activation by Aldo during AH has been better described, particularly at the experimental level. In clinical settings, patients with PA presented high sNGAL levels associated with metalloproteinase (MMP)-9 (60). However, potential modulation of sNGAL levels by MR antagonism or adrenalectomy has not been reported. Kozlowki et al. reported that uNGAL levels are increased in patients undergoing elective posterior retroperitoneal adrenalectomy; however, uNGAL was used in this case as a biomarker for kidney injury associated with the procedure and not for PA (61). This study did not report levels of sNGAL or adrenal hormones before the intervention. Therefore, new studies are needed to clarify this relationship and to support the role of sNGAL as a biomarker of PA.

At the experimental level, Latouche et al. initially demonstrated that mRNA NGAL is strongly induced in cardiac and vascular smooth muscle cells after mineralocorticoid infusion in rodents (27). However, spironolactone, an MR antagonist, prevented these effects, suggesting that the MR directly controls the NGAL expression in CV cells. Subsequently, Tarjus et al. demonstrated that high blood pressure and CV fibrosis, also characterized by galectin-3 (Gal3) and collagen (Col)-1 upregulation, induced by the “nephrectomy Aldo–salt (NAS) model,” are prevented in NGAL–KO mice, suggesting a direct link between NGAL and endocrine AH due to excess Aldo production (60). Another study showed that AH, CV fibrosis, and the pro-inflammatory phenotype in NAS mice are attenuated not only by the complete genetic deficiency of NGAL but also by depletion confined to the myeloid compartment (62). This study attained new insights regarding the effect of NGAL on APCs, considering their myeloid origin.

Recently, a study showed that Aldo induces mRNA NGAL expression in DCs by MR activation (18). In addition, CD11c^high^ APCs are necessary for developing cardiac hypertrophy and CV fibrosis in NAS mice. Buonafine et al. showed that NGAL levels were increased in macrophages (Mϕ), DCs, and peripheral blood mononuclear cells in NAS mice compared with control mice. By contrast, NGAL levels in B lymphocytes and CD4^+^ and CD8^+^ T lymphocytes of NAS-treated mice did not differ from those of control mice, even when NAS increased the recruitment of these cells in the lymph nodes (62). These findings suggest that NGAL from Mϕ and DCs is secreted during mineralocorticoid excess, which may contribute to the CV remodeling that has previously been reported. Additionally, a positive correlation was found between NGAL mRNA levels and MR in all the immune cells studied at the basal state (62), indicating a strong association between MR and NGAL in the immune cells. Notably, NGAL modulation seems specific to MR activation. Preliminary studies from our group have revealed that NGAL is not significantly modified in target CV tissues and other immune cells after stimulation of angiotensin II (AngII), another critical hormone in AH (data not published).

## The role of NGAL in the immune system during aldosterone-dependent hypertension

The link between the immune system and AH was first suggested in the 1960s, when autoantibodies were identified in the arteries of hypertensive cadavers (63). Since then, this finding has been supported by additional experimental studies (64, 65). The causal role of the immune system in secondary AH was supported by a study by Grollman and White that demonstrated that immunosuppressants could control blood pressure in rats with partial renal infarction (66). Later, additional evidence implicated the immune system in the development of AH and its association with end-organ damage (29).

Many different immune cells have been directly associated with blood pressure control. The most notable among them are monocytes/Mϕ, DCs, and T lymphocytes, which participate in AngII- or Aldo-dependent AH (18, 67–74) and NaCl-sensitive AH (75–79). Part of the mechanism proposed for T-cell activation is the formation of neoantigens from APCs (80–82) through increased reactive oxygen species formation and subsequent protein modifications (83, 84). By contrast, polymorphonuclear leukocytes, such as neutrophils, have also been considered to be among the immune cells contributing to AH (85). An increased blood neutrophil-to-lymphocyte ratio (NLR) has been observed in hypertensive patients. Moreover, one multicentric study found that NLR was positively correlated with plasma Aldo concentrations and identified it as a significant predictor of CKD among patients with PA (86). Although Aldo may directly activate the p38, ERK1/2, and PI3K pathways in human neutrophils (87, 88), which favors MMP-9/NGAL upregulation, its role in Aldo-dependent AH is still controversial, considering that Aldo inhibits nuclear factor κB (NF-κB) through MR activation in neutrophils (89). Therefore, further studies are needed to explore the Aldo–MR–NGAL relationship in neutrophil-related inflammation.

Aldo can bind to the MR in DCs and Mϕ because it is mainly present in APCs. Ko et al. demonstrated that blood pressure elevation was prevented during treatment with mineralocorticoids and salt (the DOCA–salt model) in mice deficient in Mϕ colony-stimulating factor (M-CSF), a relevant protein for Mϕ differentiation (90). Later, Rickard et al. demonstrated that myeloid MR deletion prevents AH induced by DOCA–salt. They used the Cre/LoxP recombination system, along with a lysozyme M promoter for selective MR depletion on monocytes/Mϕ (My–MR–KO), during 8 weeks of DOCA–salt treatment in mice. Cardiac remodeling associated with endocrine AH was reduced in the My–MR–KO mice (91). Subsequently, the same group demonstrated that cardiac Mϕ isolated from DOCA–salt mice showed an increment of tumor necrosis factor (TNF)-α and the chemokine C-X-C motif ligand (CXCL)-9, which promotes T lymphocyte infiltration and cardiac fibrosis, while these increases were prevented in My–MR–KO animals (92).

**Figure 1:**
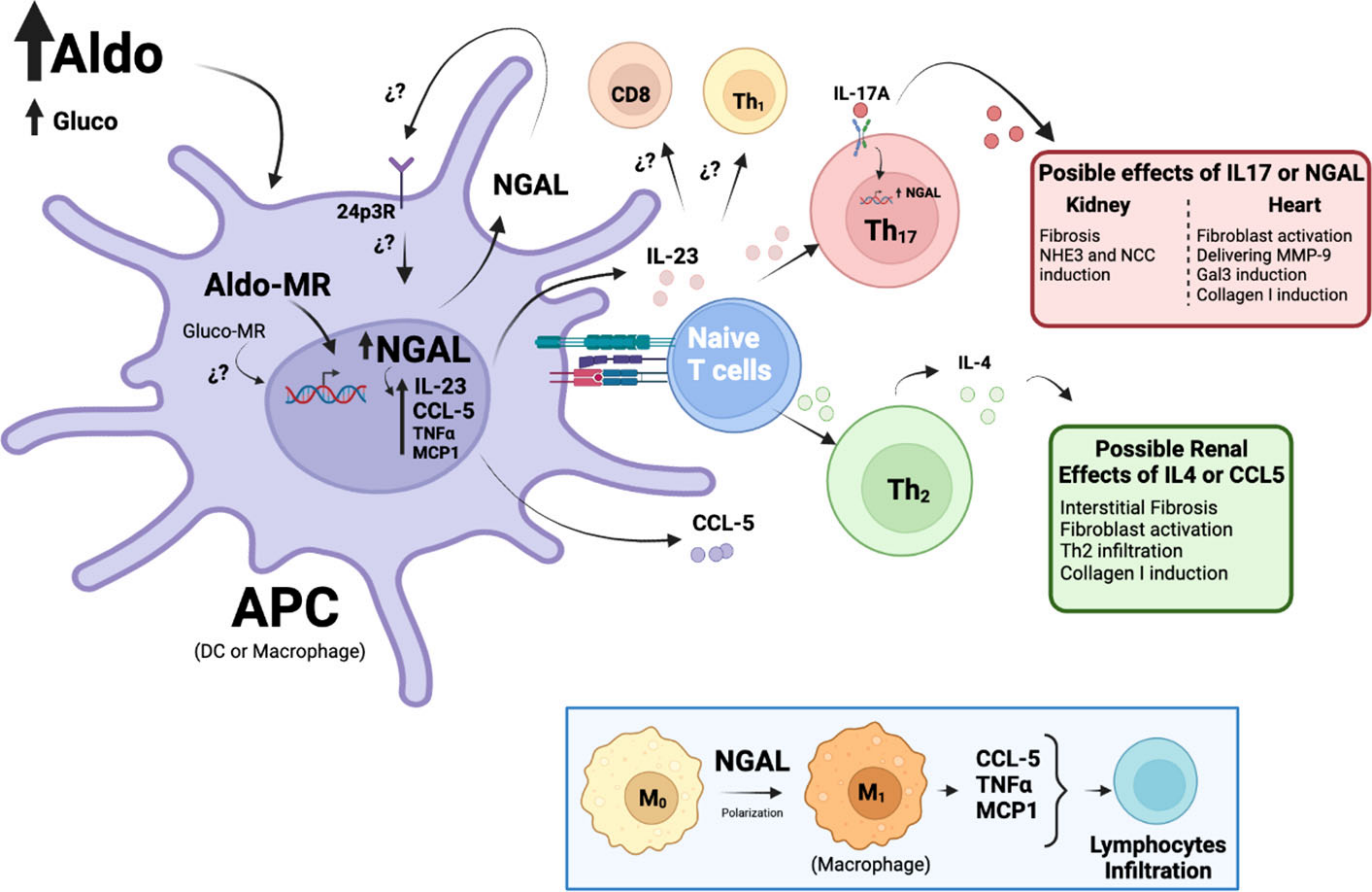
Proposed mechanisms of NGAL as an immunomodulator on APCs during Aldo-dependent endocrine hypertension. Elevated Aldo (or Gluco) levels can activate the MR in APCs (DCs or Mϕ) and promote the transcription of several genes, such as NGAL. NGAL may promote the release of pro-inflammatory factors from APCs, such as MCP-1, TNFα, and CCL-5, triggering lymphocyte infiltration in target tissues, particularly Th_2_ lymphocytes. Th_2_ lymphocytes release IL-4, favoring interstitial fibrosis and augmenting renal damage. By contrast, NGAL modulates the production of IL-23, a cytokine essential for Th_17_ lymphocyte polarization, which has been closely related to renal and CV fibrosis, as well as NHE3 and NCC induction and transcriptional increases of NGAL. Finally, NGAL may directly activate the Mϕ to M_1_ pro-inflammatory phenotype, which has also been associated with MR-dependent AH (see insert).

Based on previous evidence, the effects of the MR on APCs associated with endocrine AH can be explained by NGAL induction and its secondary impact as an immunomodulator. Recently, our group demonstrated that NGAL mediates the upregulation of IL-23p19 and IL-23p40 subunits in CD11c^high^ DCs treated with Aldo in an MR-dependent way (18). Notably, we observed that NGAL does not affect the *in vitro* differentiation of CD11c^high^ DCs from mouse bone marrow (18), which was supported by other studies in terms of maturation (93). Interestingly, IL-23p19 and IL-23p40 subunits have been reported as crucial for Th_17_ polarization from naïve T-CD4^+^ lymphocytes (94), suggesting that IL-17A secretion from Th_17_ lymphocytes may be affected by NGAL levels during hyperaldosteronism (**Figure 1**). Leopold proposed a similar mechanism in CV remodeling that considered post-translational NGAL modifications that may affect the CV abundance of Gal3 and Col-1 (95).

During kidney injury (96) and AH (97), NGAL is dependent on the IL-17A axis. Norlander et al. demonstrated that IL-17A upregulates the abundance of the sodium-hydrogen exchanger 3 (NHE3) protein and promotes the activity of sodium chloride cotransporter (NCC) in the human proximal tubule and mouse distal convoluted tubule cells, respectively (98). This suggests that NGAL may be involved in sodium reabsorption at the tubular level through IL-17A in PA. However, further studies will be needed to determine the IL-17 dependence on NGAL from APCs during MR activation.

In addition, Bonnard et al. demonstrated that NGAL from Mϕ is also necessary for the renal expression of extracellular matrix proteins, such as Col-1, αSMA, and fibronectin, which are associated with interstitial fibrosis (99). They demonstrated that NGAL is required for chemokine ligand (CCL)-5 induction in Mϕ stimulated with Aldo and salt *in vitro*, a relevant finding given that the pharmacological blockade of the CCL5 receptor reduced renal Th_2_–CD4^+^ lymphocyte infiltration induced by NAS. Finally, they observed that this blockade and the resulting neutralization of IL-4, a cytokine secreted by Th_2_-CD4^+^ lymphocytes, prevents interstitial fibrosis in the kidney, suggesting that these Th cells are also involved because of MR activation by Aldo (**Figure 1**). The previous findings indicate that NGAL may act as an immunomodulator in AH-associated PA, triggering a pro-inflammatory phenotype related to target organ damage, where the action of APCs (mainly DCs and Mϕ) is required.

Limited information is available regarding the cell types targeted by NGAL and the pathways involved. Recombinant NGAL has been shown to promote M_1_ polarization in microglia (100), which are the resident Mϕ in the central nervous system. This is relevant given that M_1_ Mϕ are responsible for producing a wide range of pro-inflammatory cytokines and chemokines, including TNF-α, CCL5, and monocyte chemokine protein (MCP)-1 (101–103). Similarly, Pawar et al. reported that 3 days of intraperitoneal injections of recombinant NGAL upregulated MCP-1 in the kidneys of wild-type mice (104). These mediators are critical for Th cell infiltration during Aldo-dependent AH, which would be modulated by NGAL (**Figure 1**, insert).

Finally, whether NGAL can activate a specific receptor on APCs and target cells at the CV and renal levels in the MR–Aldo complex remains unknown. In physiological conditions, NGAL acts mainly through its endocytic 24p3 receptor (24p3R), which is present in neurons (105), the intestinal epithelium (106), the distal and collecting tubules (107), and immune cells, such as Mϕ and neutrophils (108). However, additional studies will be needed to determine whether 24p3R is modulated by the MR–Aldo complex in the APCs involved in AH.

## Concluding remarks

Over the last 20 years, several studies have shed light on the role of the immune system in the development of AH and target organ damage, emphasizing some emerging mechanisms by which APCs activate Th cells, which has also been extensible for endocrine hypertension. Among the various origins of endocrine AH, PA seems to be the principal etiology.

Experimental studies have demonstrated that in the presence of abnormally elevated levels of Aldo, the APCs, mainly CD11c^high^ DCs and Mϕ, may potentiate additional mechanisms for antigen presentation driving T lymphocyte polarization through molecules, such as NGAL by MR activation. However, the effects of these APCs in endocrine AH, and the mechanisms by which NGAL modulates T cells, have not been fully clarified. Additional pre-clinical and clinical studies are needed to answer this question.

NGAL after MR activation in APCs would favor the differentiation and recruitment of Th_17_ and Th_2_ lymphocytes through the release of IL-23 and CCL-5, respectively. However, whether NGAL modulates polarization toward other Th lymphocytes strongly associated with AH, such as Th_1_ lymphocytes and T CD8^+^-lymphocytes, in the context of endocrine hypertension and high Aldo levels is still unknown.

## Author contributions

Conceptualization, PA and CAA; Investigation, PA and CAA; Writing—original draft preparation, PA and CAA; Writing—review and editing, CAA; Figures, PA and CAA; Supervision, CAA; Funding acquisition, PA and CAA Both authors have read and approved the published version of the manuscript.

## Funding

This work was supported by the National Agency of Research and Development (ANID), Fondecyt Regular #1201251 (CAA) and Fondecyt de Postdoctorado #3201016 (PA).

## Acknowledgments

We thank Pamela Torres for the administrative assistance at Universidad Autónoma de Chile.

## Conflict of interest

The authors declare that the research was conducted without any commercial or financial relationships that can be construed as a potential conflict of interest.

## Publisher’s note

All claims expressed in this article are solely those of the authors and do not necessarily represent those of their affiliated organizations, or those of the publisher, the editors and the reviewers. Any product that may be evaluated in this article, or claim that may be made by its manufacturer, is not guaranteed or endorsed by the publisher.
